# *Bacteroides fragilis* Protects Against Antibiotic-Associated Diarrhea in Rats by Modulating Intestinal Defenses

**DOI:** 10.3389/fimmu.2018.01040

**Published:** 2018-05-09

**Authors:** Wendi Zhang, Bo Zhu, Jiahui Xu, Yangyang Liu, Enqi Qiu, Zhijun Li, Zhengchao Li, Yan He, Hongwei Zhou, Yang Bai, Fachao Zhi

**Affiliations:** ^1^Guangdong Provincial Key Laboratory of Gastroenterology, Department of Gastroenterology, Institute of Gastroenterology of Guangdong Province, Nanfang Hospital, Southern Medical University, Guangzhou, China; ^2^Guangzhou ZhiYi Biotechnology Co. Ltd., Guangzhou, China; ^3^State Key Laboratory of Organ Failure Research, Division of Laboratory Medicine, Zhujiang Hospital, Southern Medical University, Guangzhou, China

**Keywords:** *Bacteroides fragilis*, antibiotic-associated diarrhea, gut dysbiosis, intestinal barrier function, enterocyte regeneration

## Abstract

Antibiotic-associated diarrhea (AAD) is iatrogenic diarrhea characterized by disruption of the gut microbiota. Probiotics are routinely used to treat AAD in clinical practice; however, the effectiveness and mechanisms by which probiotics alleviate symptoms remain poorly understood. We previously isolated a non-toxic *Bacteroides fragilis* strain ZY-312, which has been verified to be beneficial in certain infection disorders. However, the precise role of this commensal bacterium in AAD is unknown. In this study, we successfully established an AAD rat model by exposing rats to appropriate antibiotics. These rats developed diarrhea symptoms and showed alterations in their intestinal microbiota, including overgrowth of some pathogenic bacteria. In addition, gastrointestinal barrier defects, indicated by compromised aquaporin expression, aberrant tight junction proteins, and decreased abundance of mucus-filled goblet cells, were also detected in ADD rats compared with control animals. Of note, oral treatment with *B. fragilis* strain ZY-312 ameliorated AAD-related diarrhea symptoms by increasing the abundance of specific commensal microbiota. Interestingly, we demonstrated that these changes were coincident with the restoration of intestinal barrier function and enterocyte regeneration in AAD rats. In summary, we identified a potential probiotic therapeutic strategy for AAD and identified the vital roles of *B. fragilis* strain ZY-312 in modulating the colonic bacterial community and participating in microbiota-mediated epithelial cell proliferation and differentiation.

## Introduction

Antibiotic-associated diarrhea (AAD) refers to iatrogenic diarrhea associated with antibiotic therapy and most frequently affects in-patients, especially the elderly (≥65 years), treated with broad-spectrum antibiotics (1). The clinical manifestations of AAD usually include mild and self-limiting diarrhea. It has been reported that 15–39% of AAD cases are caused by excessive growth of opportunistic pathogens, including *Clostridium difficile*, which can result in pseudomembranous colitis or a toxic megacolon, contributing to high fatality rates (2). In addition, diarrhea triggered by antibiotic use is associated with impaired resistance to pathogens as a result of the disruption of the gut microbial flora and subsequent changes in the metabolism of carbohydrates, short-chain fatty acids, and bile acids (3).

Probiotics are defined as live microorganisms that confer health benefits to the host when administered in adequate amounts (4). As AAD results from disruption of the commensal gut microbiota due to antibiotic therapy, administration of probiotics is a reasonable therapeutic strategy to regulate or restore the gut microbiota (5). A previously published meta-analysis showed that most probiotics significantly reduce the risk of AAD in the general (mainly adult) population (6). These impressive effects have motivated many healthcare institutions to consider routine probiotic co-administration along with antibiotic treatments. However, there is still controversy regarding the usefulness of routine probiotic administration. Results of the PLACIDE trial, the largest randomized controlled trial (*n* = 2941) to date and conducted across five centers in England and Wales, showed that 21 days of treatment with a combined preparation of *Lactobacillus* and *Bifidobacterium* did not reduce the risk of either AAD or *C. difficile*-associated diarrhea (7). However, because the study was limited to only two bacterial genera among the multitude of non-pathogenic, potentially beneficial bacteria, definitive conclusions cannot be drawn regarding the benefits of probiotics for the treatment or prevention of AAD—that is, these two bacteria may not be sufficient to tip the balance of a diverse gut ecosystem, and other taxa or probiotic mixtures may be beneficial (8).

Gram-negative *Bacteroides* species are among the earliest colonizing and most abundant constituents of the gut microbiota (9). Among the *Bacteroides* species, *Bacteroides fragilis* is an important obligate anaerobe that colonizes the mammalian lower gastrointestinal tract (10). Two subtypes of *B. fragilis* have been identified and are referred to as non-enterotoxigenic *B. fragilis* (NTBF) and enterotoxigenic *B. fragilis* (ETBF). Recent studies identified the pathogenicity of ETBF with the ability to cause diarrheal disease in animals, children, and adults (11). Conversely, NTBF strains have been proposed as possible probiotics, with the potential to quell colonic inflammation. Mazmanian et al. (12–14) conducted a series of studies to demonstrate that polysaccharide A produced by *B. fragilis* strain NCTC 9343 induces an anti-inflammatory milieu involving the stimulation of interleukin-10-producing CD4^+^Foxp3^+^ T-regulatory cells in the intestine, thereby reducing pathological gastrointestinal symptoms in a mouse model of colitis. They also suggested the therapeutic potential of *B. fragilis* strain NCTC 9343 for autism spectrum disorder, whereby alteration of the gut permeability and microbial composition may ameliorate related behavioral abnormalities (15). Kasper et al. (16) also showed that *B. fragilis* strain NCTC 9343 can protect against neuroinflammation in mouse models of multiple sclerosis. Collectively, these studies raised the possibility that non-enterotoxigenic *B. fragilis* may be crucial for the establishment of beneficial intestinal microbiota and could be developed into a probiotic therapy.

We previously isolated NTBF *B. fragilis* strain ZY-312 (ZY-312) from the feces of a healthy, breast-fed infant and confirmed that it does not contain any potential virulence factors or transferable antibiotic resistance genes (17, 18). We hypothesized that this strain may alleviate AAD-related gastrointestinal symptoms and correct any associated gut ecosystem abnormalities, thereby representing a promising candidate for the prevention and/or treatment of AAD. To test this hypothesis, a stable and reproducible model of AAD must first be established. Most previous AAD models have been induced by certain pathogenic bacteria (19). However, given that *C. difficile*-associated diarrhea only accounts for 10–25% of AAD cases, we established a novel AAD rat model induced by repeated oral administration of ampicillin, streptomycin, and clindamycin. This model effectively induced the development of soft or watery stools in rats, as well as defects in intestinal integrity and alterations in the composition of commensal microbiota. We used this model to examine the therapeutic effects of ZY-312 on AAD and to explore the underlying mechanisms. Specifically, the bacterial composition and diversity in the gut were examined by 16S rRNA gene sequencing and bacterial culture in model rats gavaged with various doses of ZY-312, commercial probiotic Bifico, or a combination of Bifico and ZY-312. In addition, gastrointestinal barrier function was assessed at multilevels by quantitative real-time polymerase chain reaction assays (qRT-PCR), western blotting, immunofluorescence, and immunohistochemistry. The results of this study confirm the potential application of *B. fragilis* ZY-312 as a novel probiotic for patients with AAD.

## Materials and Methods

### Ethics Statement

All animal experiments were approved by Nanfang Hospital Animal Ethics Committee (protocol # NFYY-2014-123), in accordance with relevant ethical principles and guidelines set by the Animal Welfare Act and the NIH Guide for the Care and Use of Laboratory Animals. Experiment involving isolation of *B. fragilis* strain ZY-312 from infant fecal was approved by the Medical Ethics Committee of NanFang Hospital (NFEC-2014-040).

### Animals and the AAD Model

Sprague-Dawley rats weighing 230–250 g were purchased from the Experimental Animal Center of Southern Medical University (Guangzhou, China) and housed under specific pathogen-free conditions. The AAD model was induced by gavage daily with 2 ml of normal saline containing a mixture of clindamycin (Hisoar Pharmaceutical, Zhejiang, China), ampicillin (Jianmin Pharmaceutical Group Co., Ltd., Wuhan, China), and streptomycin (Lukang Pharmaceutical Co., Shandong, China) at various doses: low dose (combination of 25 mg/ml clindamycin, 27.75 mg/ml ampicillin, and 13.88 mg/ml streptomycin); middle dose (combination of 50 mg/ml clindamycin, 55.5 mg/ml ampicillin, and 27.75 mg/ml streptomycin); high dose (combination of 75 mg/ml clindamycin, 83.25 mg/ml ampicillin, and 41.63 mg/ml streptomycin). The antibiotics were gavaged for 7 days, and the doses were determined to be 1.35, 2.70, and 4.05 times the maximum human equivalent dose, respectively, based on previous studies (20, 21). Weight, water intake, and the presence of diarrhea were measured every second day during the experimental period. Fecal samples were collected every 3 days for microflora analyses. On days 8 and 14, half of the rats in each group were euthanized, and 2-cm-long samples of colon tissue 3-cm distal to the anus were collected for general histopathological analyses.

### Diarrhea Assessment

**Figure 1:**
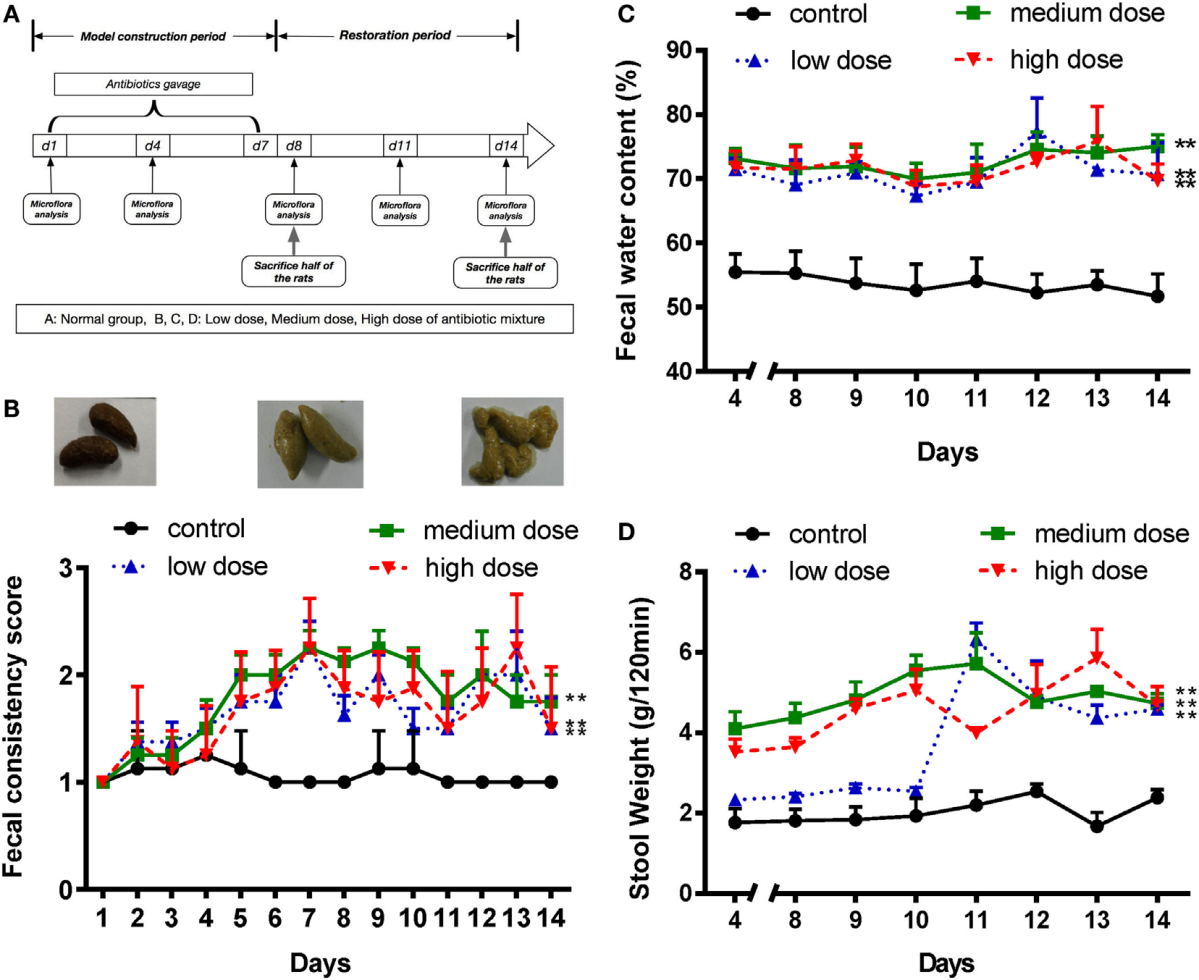
An appropriate mixture of antibiotics induces diarrhea in SD rats. **(A)** A schematic of antibiotic-associated diarrhea model. Rats were gavaged daily with antibiotics mixture for 7 days. **(B)** Fecal consistency was assessed and classified by three visual grading scale (upper panels), and fecal consistency scores were shown in all different groups (lower panel). **(C,D)** Fecal water content and 120-min stool weight were measured in rats treated with normal saline or different doses of antibiotic cocktails (low, medium, and high), from day 4 to day 14, *n* = 8/group. Repeated measures ANOVA, *P < 0.05 and **P < 0.01 versus normal group.

Diarrhea symptoms were assessed using three parameters: fecal consistency, fecal output weight in 120 min, and fecal water content, as described previously (19, 22). Fecal consistency was classified based on the following visual grading scale: (1) formed, stool maintains its shape, brown, score = 1 (Figure 1B, upper left panel); (2) semi-formed or soft, does not pour, yellow, score = 2 (Figure 1B, upper middle panel); and (3) liquid, pours more easily, yellow, score = 3 (Figure 1B, upper right panel). Fecal output was determined by measuring cumulative stool weight (mg) over a 120-min period. Fecal samples were weighed and dried, and then the dried solid content and total fecal content were measured. The fecal water content was calculated as follows: fecal water content = 1 − (dried solid content)/(total fecal content).

### Bacteria Culture

*Bacteroides fragilis* strain ZY-312 was isolated from the feces of a healthy, breast-fed infant as reported (17). And the safety evaluation of ZY-312 was done previously (18). Bacteria were cultured in sterile tubes containing 10 ml of tryptone soy broth supplemented with 5% fetal bovine serum and incubated anaerobically at 37°C for 24 h in an anaerobic cabinet (Mart, Drachten, The Netherlands).

### Live Bacterial Culture

Fresh feces were homogenized (10% w/v) and serially diluted in *Brucella* broth (Hope Biotechnology, Qingdao, China). Aliquots were then spread onto different selective agar plates (10^−2^ to 10^−9^ dilutions). Mannitol salt agar, BBE agar, TPY medium (Hope Biotechnology), eosin-methylene blue medium (Oxoid, Hampshire, UK), and CDC Anaerobic Agar were used to detect *Staphylococcus aureus, Bacteroides, Bifidobacterium*, Enterobacteriaceae, and *Peptococcus*, respectively. *Lactobacillus* selective agar supplemented with 1.32 ml/l glacial acetic acid was used to select *Lactobacillus* species, while CATC agar and DRBC agar were used to isolate fecal *Enterococcus* and Saccharomycetes. Reinforced *Clostridium* agar supplemented with egg was used for *Clostridium* detection. The plates were incubated at 37°C in an anaerobic cabinet or aerobic atmosphere as appropriate for 48 h.

### Treatment of AAD Rats With *B. fragilis* ZY-312

During the treatment period, AAD rats were gavaged daily with 2 ml of sterile normal saline containing three different doses of *B. fragilis* ZY-312 [10^7^, 10^8^, or 10^9^ colony-forming units (CFU)], Bifico (70 mg, SINE, Shanghai, China, containing 10^8^ CFU of freeze-dried *Bifidobacterium longum, Lactobacillus acidophilus*, and *Enterococcus faecalis*), or a combination of Bifico and *B. fragilis* ZY-312 (10^8^ CFU). A placebo group was gavaged with saline, while healthy, untreated animals served as the normal control group. For quality control, collection and administration of bacteria was finished within half an hour.

Following pre-challenge with the antibiotic mixture for 7 days (day 1–7), rats were treated as described above for 4 days (day 8–11) or 7 days (day 8–14). Diarrhea symptoms, body weight, and water intake were recorded daily. On day 11 (in the middle of treatment) and day 17, half of the animals were euthanized, respectively. Colons were collected as described above, and dissected longitudinally using sterile tissue scissors. Fecal matter (~200 mg) from the colon of each rat was then collected in 1.5-ml sterile tubes and frozen at −80°C until DNA extraction.

### Microbial DNA Extraction and 16S rRNA Gene Sequencing

Rat fecal microbial DNA was extracted from stool samples using a QIAamp DNA Mini Kit (Cat.: 51504, QIAGEN, Hilden, Germany) following the manufacturer’s instructions. The V3–V4 region of the 16S rRNA gene was then amplified from fecal DNA samples using the 341F/805R primer pair (341F: 5′-CCTACGGGNGGCWGCAG-3′, 805R: 5′-GACTACHVGGGTATCTAATCC-3′). All libraries were sequenced using the Illumina MiSeq platform (Illumina, San Diego, CA, USA) by Huayin Co., Guangdong, China.

Sequence analysis was performed in the Quantitative Insights into Microbial Ecology (QIIME, release v. 1.9.1) framework as follows (23). The 16S rRNA reads were initially screened for low quality bases and short read lengths. Paired reads were filtered for quality (Q30), and then assembled using Fast Length Adjustment of Short reads (v1.2.11). After filtering, from a total of 84 fecal samples, we obtained a total of 3,777,000 high-quality sequences. The average number of reads per sample was 44,965 (±2,992 SD). The resulting consensus sequences were de-multiplexed (i.e., assigned to their original sample) and trimmed of artificial barcodes and primers. Chimeric sequences were identified and removed using the usearch61 algorithm against the GreenGenes database (v13_8). We used the QIIME command pick_closed_reference_otus.py to cluster the clean data into operational taxonomic units (OTUs), and obtained a biom-format OTU table. In this process, sequences were clustered into OTUs by Usearch61_ref (v5.2.236) based on a 97% similarity threshold with the GreenGenes database (v13_8). The Chao diversity index and the number of observed species per sample were used as α-diversity metrics. β-diversity was calculated using unweighted UniFrac distances and represented in principal coordinate analyses (PCoA). Diversity was calculated by QIIME, and analyses were performed and visualized in R (v3.2.2) using pheatmap and ade4 packages. Linear discriminant analysis effect size was used to detect unique biomarkers (LDA score >3.5) in relative abundance of bacterial taxonomy (24).

Sequence files for all samples used in this study have been deposited in the European Nucleotide Archive under the accession number PRJEB22950, at http://www.ebi.ac.uk/ena/data/view/PRJEB22950. Analysis scripts and relevant files are available in GitHub (https://github.com/SMUJYYXB/B.fragilis_AAD).

### Histological Analysis

Colon tissues were fixed in 4% (w/v) PFA and embedded in paraffin. Thereafter, 4-mm sections were cut and stained with hematoxylin–eosin. Colitis was assessed in tissue sections and was scored by a pathologist using a blinded experimental setup according to a standard scoring system: 0, no thickening of the colonic tissues and no inflammation (infiltration of lymphocytes); 1, mild thickening of tissues, but no inflammation; 2, mild thickening of tissues and mild inflammation; and 3, severe thickening and severe inflammation (13).

For goblet cell counting, the deparaffinized tissue sections were incubated in a 3% (v/v) glacial acetic acid solution for 3 min, in an alcian blue solution for 15 min, and then stained with Nuclear Fast Red (Sigma) for 5 min. Goblet cells were quantified by counting the numbers of alcian blue-positive cells in cross-sectional views of 30 colonic crypts per rat.

For immunofluorescence staining, deparaffinized tissue sections were steamed in citrate buffer for antigen retrieval, and blocked using phosphate-buffered saline (PBS) containing 1% (w/v) bovine serum albumin (BSA). The rabbit-derived primary antibody anti-ZO-1 (Cat.: 402200; ThermoFisher Scientific, Carlsbad, CA, USA) was used at a dilution of 1:100, and the secondary goat anti-rabbit antibody conjugated to AlexaFluor 488 was used at a dilution of 1:2,000.

For immunohistochemistry staining, the paraffinized sections were deparaffinized, rehydrated, and heat-induced antigen retrieval was performed in citrate buffer. PBS containing 1% (w/v) BSA was used to block the tissues. Primary antibodies anti-MUC2 (Cat.: 134119; Abcam), anti-Ki67 (Cat.: 12202s; CST), and anti-p-extracellular signal-related kinase (ERK) (Cat.: 4370; CST) were used at dilutions of 1:100, 1:500, and 1:200, respectively. Biotinylated secondary antibodies were purchased from ZSGB-BIO (China). Staining was visualized using 3,3′-diaminobenzidine substrate.

All samples were imaged using an Olympus fluorescence microscope (BX53; Tokyo, Japan).

### Tissue RNA Extraction and qRT-PCR Assays

Total RNA was prepared from the colon tissue using TRIzol reagent (Takara, Shiga, Japan) as per the manufacturer’s instructions. cDNA was generated from 1 µg of total RNA using a PrimeScript RT Reagent Kit and gDNA Eraser (Takara). Primers were designed and synthesized as previously described (5) by Sangon Biotech (Shanghai, China), and primer sequences are provided in Table S2 in Supplementary Material. qRT-PCR assays were carried out using SYBR green I Master (Roche, Basal, Switzerland) under the following conditions: 95°C for 15 s, 94°C for 10 s, 60°C for 30 s, and 72°C for 30 s, repeated for 45 cycles.

### Western Blotting

Rat colon tissues were lysed using radioimmunoprecipitation assay lysis buffer (Beyotime, Haimen, China), and tissue lysates were centrifuged at 14,000 × *g* for 30 min. Supernatants were collected and mixed with 5× sodium dodecyl sulfate (SDS) sample buffer. The samples were separated by SDS-polyacrylamide gel electrophoresis using 8–12% acrylamide gels, and then transferred to polyvinylidene fluoride membranes (Millipore, Billerica, MA, USA). Following incubation with primary and secondary antibodies, protein bands were detected with Immobilon Western Substrate (Millipore) and analyzed using the Bioimage analysis system (Syngene, Frederick, MD, USA). The following antibodies were used: rabbit anti-ZO-1, rabbit anti-occludin (Cat.: ab31721; Abcam), rabbit anti-phospho-ERK, rabbit anti-ERK (Cat.: 4695; CST), rabbit anti-phospho-P38 (Cat.: 4511S; CST), rabbit anti-P38 (Cat.: 8690; CST), rabbit anti-phospho-c-Jun N-terminal kinase (JNK) (Cat.: 4668; CST), and rabbit anti-JNK (Cat.: 9252; CST).

### Statistical Analysis

Data are presented as means ± SEMs, unless otherwise indicated. All experiments were performed in triplicate or greater, and data are representative of five or more independent experiments. Statistical analysis of significant differences was performed using a two-tailed *t*-test, repeated measurement analysis of variance, or one-way analysis of variance, where appropriate. *P*-values of <0.05 were considered statistically significant.

## Results

### Disruption of the Intestinal Microbiota by a Mixture of Antibiotics Triggers Diarrhea in Rats

Various doses of antibiotic mixture containing clindamycin, ampicillin, and streptomycin were used to induce AAD in a rat model (Figure 1A). Compared with the control group, the AAD groups showed no significant differences in body weight or food intake (Figures S1A,B in Supplementary Material). However, water intake increased significantly (*P*<0.01) in the AAD groups in a dose-dependent manner (Figure S1C in Supplementary Material). Notably, rats with AAD exhibited obvious diarrhea, as measured by fecal consistency, fecal water content, and 120-min stool weight (22, 25). Loose or liquid stools were first observed in the AAD rats on day 4, with symptoms most obvious on days 7 and 8, and lasting up to day 14 (Figure 1B). In addition, the fecal water content substantially increased in all AAD groups from day 4 to day14 (Figure 1C). The 120-min stool weight increased steadily in the medium dosage group and was significantly higher than that of the control group after day 4. In the high dosage group, parameter values fluctuated dramatically, while no significant difference was observed for the low dosage group until day 11 (Figure 1D). These results indicated that long-term gavage of antibiotics caused diarrhea in rats, although the severity of symptoms was not dose-dependent. Furthermore, histological examination of the intestinal tissues revealed no obvious inflammation in rats from any of the antibiotics-challenged groups (Figures S1D–F in Supplementary Material).

**Figure 2:**
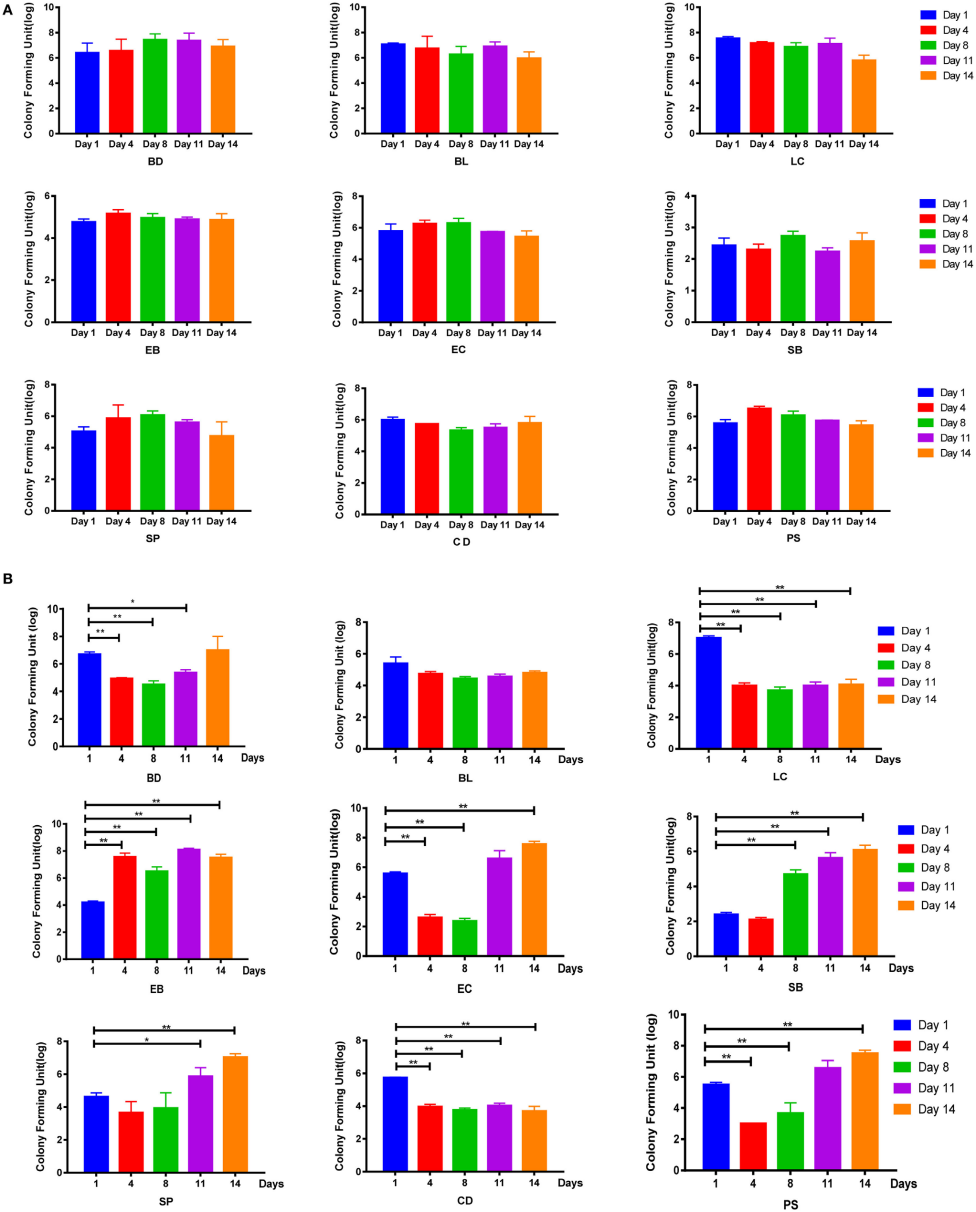
Disruption of SD rats microbiota by a mixture of antibiotics. **(A,B)** Bacterial culture for microbial community composition analysis of normal control rats and antibiotic-associated diarrhea rats induced by the medium dose of antibiotic cocktail. Abbreviations: BD, *Bacteroides* (in BBE agar); BL, *Bifidobacterium* (in TPY medium); LC, *Lactobacillus* (in *Lactobacillus* selective agar); EB, *Enterobacteriaceae* (in eosin-methylene blue medium); EC, *Enterococcus* (in CATC agar); SB, Saccharomycete (in DRBC agar); SP, *Staphylococcus aureus* (in Mannitol salt agar); CD, *Clostridium* (in reinforced *Clostridium* agar); PS, *Peptococcus* (in CDC anaerobic agar). *t*-Test, *P < 0.05 and **P < 0.01 versus day 1.

To examine whether these symptoms were associated with the disruption of intestinal flora, bacterial culture-based assays were performed. Results showed that the intestinal flora of the control group remained relatively stable throughout the experimental period (Figure 2A; Figure S2A in Supplementary Material). By contrast, in the AAD groups, decreases in the abundance of some bacterial genera, including *Lactobacillus* and *Clostridium*, were observed on day 4, and these populations had not recovered by day 14. In addition, *Bacteroides, Enterococcus, S. aureus*, and *Peptococcus* were inhibited on day 4, but these populations recovered to their pre-challenge levels (or even higher) by day 11. Interestingly, increases in the abundance of Enterobacteriaceae and Saccharomycetes were observed in the AAD groups on days 4 and 8, respectively, and these populations were still present at increased levels on day 14 (Figures 2B; Figure S2B in Supplementary Material). However, there were no statistically significant differences among groups treated with different doses of the antibiotics. Together, these findings indicated that the mixture of antibiotics did cause disruption of the intestinal microbiota. These changes fluctuated over time, and had not been restored to their pre-challenge levels by day 14.

These findings suggest that disruption of the microbiota by a mixture of antibiotics makes conventional rats susceptible to AAD. As a dose-dependent effect of the antibiotics was not evident, we used the medium dose of antibiotic cocktail to induce AAD in subsequent assays.

### *B. fragilis* ZY-312 Treatment Reduces AAD-Related Gastrointestinal Symptoms

**Figure 3:**
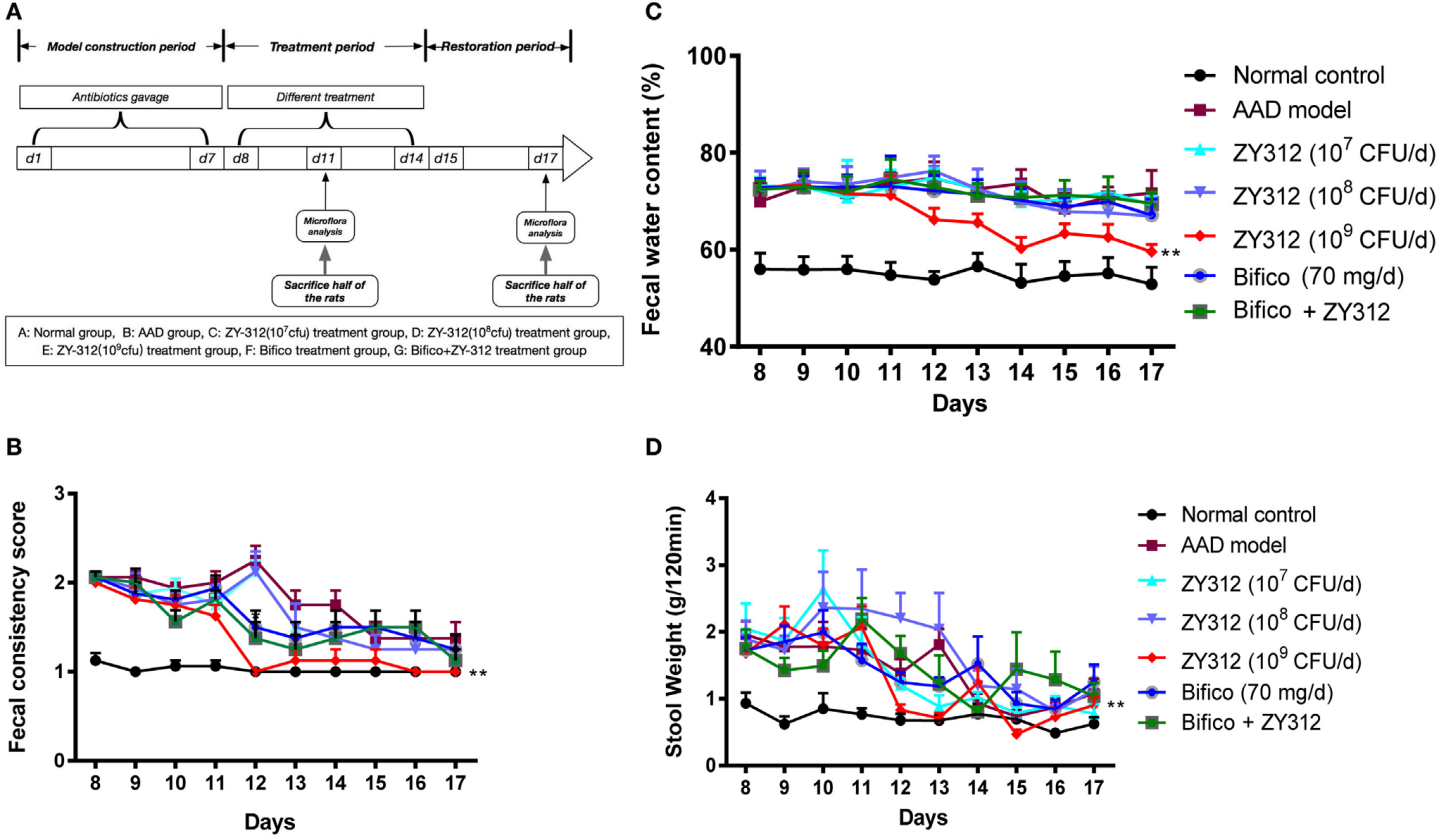
*Bacteroides fragilis* ZY-312 improves antibiotic-associated diarrhea (AAD)-related gastrointestinal symptom. **(A)** Experimental design schematic of different treatments in AAD rats. Rats were pretreated with antibiotic mixtures for 7 days, followed by bacterial treatment. During the treatment period (day 8–14), rats were gavaged with normal saline, ZY-312 [10^7^, 10^8^, and 10^9^ colony-forming units (CFU)/day], Bifico (70 mg/day), or Bifico (70 mg/day) combined with ZY-312 (10^8^ CFU). **(B–D)** Fecal consistency scores, fecal water content, and 120-min stool weight of rats in each group from day 8 to day 17 were shown. *n* = 16/group. Repeated measures ANOVA, *P < 0.05 and **P < 0.01 versus AAD group.

To test the effects of *B. fragilis* ZY-312 treatment in the rat AAD model, we treated AAD rats with various doses of ZY-312, Bifico, or a combination of Bifico and ZY-312. A schematic diagram of the experimental design is shown in Figure 3A. There were no significant differences in weight or food intake among groups (data not shown). However, while water intake remained elevated in most groups, the water intake of AAD rats treated with 10^9^ CFU of ZY-312 returned to normal levels on days 12 and 13 (Figure S3A in Supplementary Material). Most rats in the AAD group treated with 10^9^ CFU of ZY-312 produced formed, solid stools after day 12, and the fecal consistency score in this group decreased significantly (*P* < 0.01) compared with the AAD group. Similar results were not observed in the other treatment groups or the AAD group until day 14 (Figure 3B). In line with the fecal consistency results, fecal water content in the AAD group treated with 10^9^ CFU of ZY-312 significantly decreased (*P* < 0.01) compared with the AAD group after day 12, while the fecal water content of other treatment groups was not significantly reduced (Figure 3C). Similarly, the 120-min stool weight in the 10^9^ CFU ZY-312 treatment group decreased by day 12, while those of other groups were not reduced until day 14 (Figure 3D).

Therefore, treatment with a sufficient amount of *B. fragilis* ZY-312 appeared to have a protective effect in AAD rats, which encouraged us to further investigate the mechanism(s) underlying these effects.

### *B. fragilis* ZY-312 Treatment Modulates Specific Microbial Changes in AAD Rats

**Figure 4:**
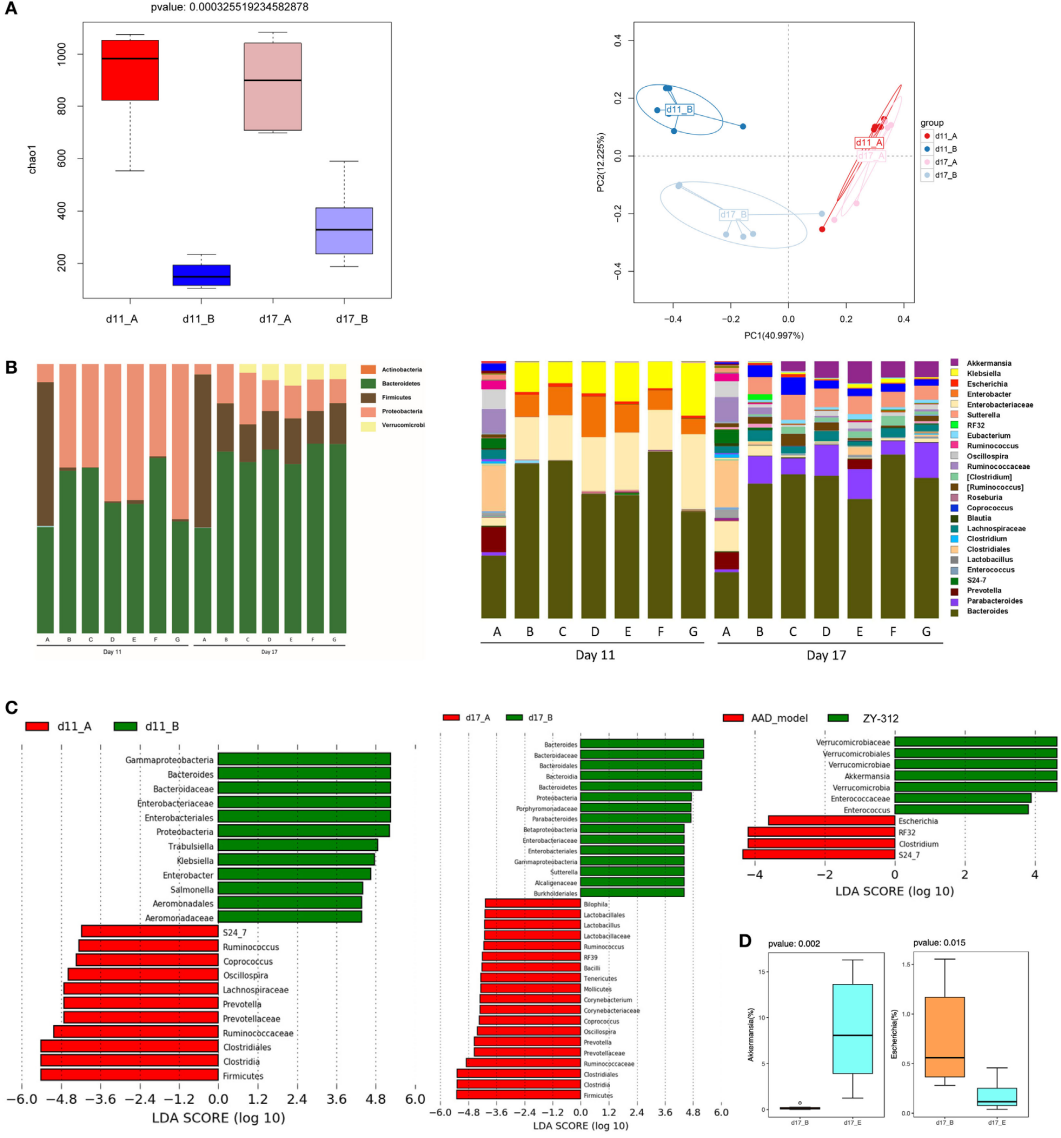
*Bacteroides fragilis* ZY-312 restores specific microbiota changes in antibiotic-associated diarrhea (AAD) rats. **(A)** Microbial richness based on the Chao1 index, and unweighted UniFrac principal coordinate analyses based on all operational taxonomic units (OTUs) of normal rats (group A) and AAD rats (group B) on days 11 and 17, respectively. **(B)** Relative abundance of OTUs of the gut microbiota in all groups, including normal control (group A) and AAD groups gavaged with normal saline (group B), different doses of ZY-312 (10^7^, 10^8^, and 10^9^ CFU) (group C, D, and E), Bifico (70 mg/day) (group F), or Bifico (70 mg/day) combined with ZY-312 (10^8^ CFU) (group G) by gavage, at the rank of phylum (left panel) and genus (right panel). **(C)** Linear discriminant analysis effect size identifies differential abundance of bacteria between normal rats (group A) and AAD rats (group B) on days 11 and17, or between AAD rats (group B) and ZY-312 (10^9^ CFU) rats (group E) (LDA > 3.5), respectively. **(D)** Relative abundance of OTUs of the genus *Akkermansia* and *Escherichia* that are significantly altered by ZY-312 treatment (group E).

As dysbiosis of the intestinal microbiota is a characteristic of AAD, we next investigated whether *B. fragilis* ZY-312 modulates the composition of the microbial community, thereby providing a protective effect against AAD. 16S rRNA gene sequencing was used to evaluate specific microbial alterations in all groups on days 11 and 17. Based on estimates of alpha-diversity, the total bacterial community in AAD rats was decreased on day 11 compared with the normal control group, but recovered slightly by day 17 (Figure 4A, left panel). In addition, unweighted UniFrac analysis revealed that the overall composition of the fecal microbial community of AAD rats was significantly different from that of normal controls on day 11, but that the difference between both groups appeared to be smaller on day 17 than on day 11 (Figure 4A, right panel). However, no significant differences were observed in the evenness or richness among groups of ZY-312-treated or Bifico-treated AAD rats (Figure S4A in Supplementary Material). Unweighted UniFrac analysis also demonstrated that none of the treatment groups clustered distinctly from the AAD group on either day 11 or day 17 based on PCoA (Figure S4B in Supplementary Material).

We also evaluated differences in the relative abundance of different taxa among groups. On day 11, the AAD group gut microbiota was mainly dominated by Proteobacteria, while the predominant phylum in control rats was Firmicutes. In addition, the bacterial community composition in the AAD rats had not reverted to the control composition by day 17. Interestingly, although we did not detect obvious differences among the AAD group and the treatment groups on day 11, there was a significant increase in the relative abundance of the phylum Verrucomicrobia in the treatment groups on day 17 (Figure 4B, left panel).

At the genus level, four genera of beneficial microorganisms, *Oscillospira, Prevotella, Ruminococcus*, and *Coprococcus*, which include species involved in short-chain fatty acid production, were decreased or even eliminated in AAD rats on day 11, and had not been restored to control group levels on day 17. The genera *Enterobacter, Klebsiella, Trabulsiella, Salmonella*, and *Escherichia*, all of which demonstrate pathogenic characteristics, were enriched in AAD rats on day 11. By day 17, the relative abundance of the genera *Enterobacter, Salmonella*, and *Klebsiella* had returned to levels similar to the control, although there was an increase in the abundance of *Sutterella* (Figure 4B, right panel and Figure 4C). In addition, although observed changes in the abundance of *Oscillospira, Ruminococcus, Coprococcus, Klebsiella*, and *Enterobacter* in the treatment groups were similar to those of AAD rats, and no significant differences were found among treatment groups (Figures 4B,C, right panels), our findings showed that one beneficial microorganism, *Akkermansia*, belonging to the phylum Verrucomicrobia, increased significantly (*P* = 0.002) in the 10^9^ CFU ZY-312 treatment group on day 17, while a significant decrease (*P* = 0.015) was observed in the abundance of *Escherichia* (Figure 4C, right panel and Figure 4D).

Together, these results demonstrate that dramatic overgrowth of *Klebsiella* and *Enterobacter*, and loss of *Oscillospira, Prevotella*, and *Ruminococcus* species, may play a role in AAD pathogenesis. The results also confirmed that *B. fragilis* ZY-312 can modulate specific components of the commensal microbiota in AAD rats.

### *B. fragilis* ZY-312 Improves Gut Barrier Integrity in AAD Rats

**Figure 5:**
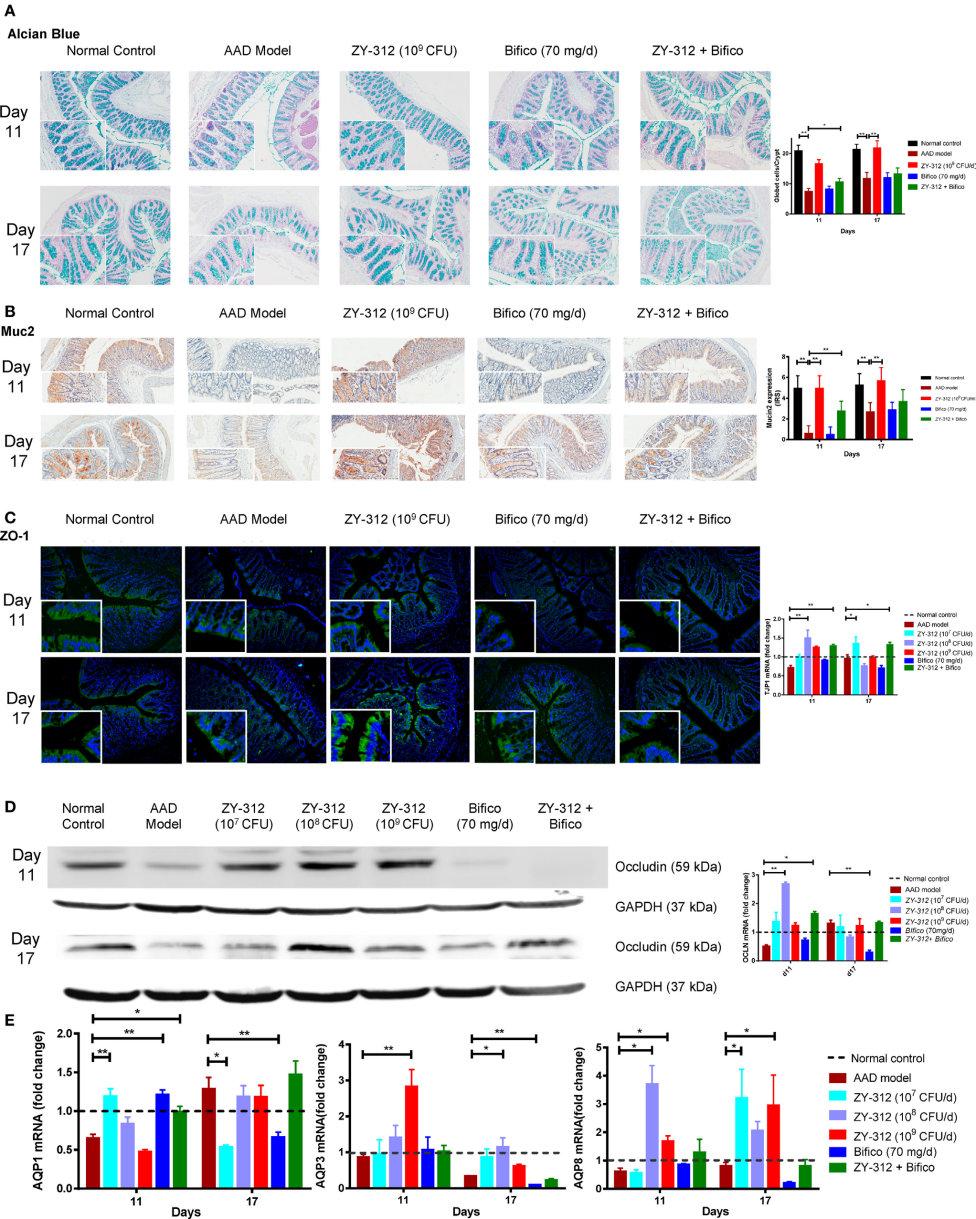
*Bacteroides fragilis* ZY-312 improves gut barrier integrity in antibiotic-associated diarrhea (AAD) rats. **(A)** Alcian blue staining for detecting goblet cells numbers were shown in normal rats, AAD rats treated with normal saline, ZY-312 [10^9^ colony-forming units (CFU)], Bifico (70 mg/day), or Bifico (70 mg/day) combined with ZY-312 (10^8^ CFU) on days 11 and 17. *n* = 8/group. **(B)** Immunohistochemistry of Muc2 protein located in colonic tissues was detected in all groups as mentioned above. **(C)** Immunofluorescence staining for ZO-1 and *ZO-1* (*TJP1*) mRNA level in all the groups was shown as mentioned above. The magnifications of the above figures are 10× and 40×. **(D)** Colon protein level and mRNA expression of occludin in all groups on days 11 and 17 were shown. **(E)** qPCR analysis of AQP1, AQP3, and AQP8 gene in colonic tissues were detected in all groups as indicated in Figure 3, with mRNA fold changes normalized to those of normal control group. One-way ANOVA, *P < 0.05 and **P < 0.01 versus AAD group.

The intestinal epithelium is the interface between the gut microbiota and host tissues and is an important physical barrier preventing luminal pathogens from entering the blood stream. Histologic examination showed no obvious damage or inflammation of the intestinal epithelium in any of the experimental groups (Figure S3B in Supplementary Material). We next analyzed epithelial cell integrity by examining the expression of aquaporin and tight junction proteins in the different groups to identify possible histological and molecular changes related to diarrhea. As shown in Figure 5A, there were twofold and onefold decreases in the number of mucus-filled goblet cells in the colon on days 11 and 17, respectively, indicating that the secretion of mucus from goblet cells may be reduced in the colons of AAD rats. Treatment with 10^9^ CFU of ZY-312 resulted, on average, in a onefold increase in goblet cell numbers in the colon on both days 11 and 17 compared with the AAD group. By contrast, other treatment groups showed no significant differences. Consistent with the above-described changes, the expression of *muc2*, which is involved in the synthesis and secretion ability of goblet cells, was significantly lower in the colons of AAD rats than in control rats on day 11, and had only slightly recovered on day 17. However, the expression of *muc2* was increased in the colons of 10^9^ CFU ZY-312-treated rats on both days 11 and 17. By contrast, Bifico alone or in combination with a medium dose of ZY-312 (10^8^ CFU) had no significant effect on *muc2* expression compared with the AAD group (Figure 5B).

We next assessed the localization and expression of ZO-1 and occludin in the colon by immunofluorescence or western blotting. Compared with the control group, the arrangement of the tight junction protein ZO-1 in AAD rats was aberrant, with discontinuous focal accumulation and reduced staining intensity in the lateral membrane on both days 11 and 17. In the ZY-312 treatment group (high dose), ZO-1 was localized to the apical regions, with intact boundaries, on days 11 and 17, as evidenced by enhanced ZO-1 immunostaining in the colon compared with that in AAD rats. However, Bifico alone failed to correct the aberrant ZO-1 expression (Figure 5C, left panel). Western immunoblotting results confirmed that the colonic epithelial cells of AAD rats had reduced levels of occludin, but that treatment with a high dose of ZY-312 resulted in an increase in occludin on days 11 and 17 (Figure 5D, left panel). In line with the protein expression results, the levels of *ZO-1* (*TJP1*) and *occludin* mRNA in AAD rats were decreased to 72 and 51%, respectively, of the levels observed in controls rats on day 11. Surprisingly, the 10^8^ CFU ZY-312 treatment group showed significantly enhanced *ZO-1* and *occludin* mRNA levels compared with AAD rats on day 11, while there was only a slight increase in the transcription of these genes in the 10^9^ CFU ZY-312 treatment group. Bifico did not appear to affect the transcriptional levels of either *ZO-1* or *occludin*. The expression levels of the genes encoding *ZO-1* and *occludin* in AAD rats were similar to those of the controls on day 17, and there was no significant difference between the treatment groups and the AAD group at this time point (Figures 5C,D, right panels).

We also investigated the expression levels of genes encoding aquaporins (*Aqp*) in the colon and demonstrated that the expression of *Aqp1, Aqp3*, and *Aqp8* in AAD rats was reduced to 65, 86.2, and 61.1% of the levels in control rats, respectively, on day 11. On day 17, *Aqp3* and *Aqp8* mRNA levels in the AAD rats remained lower than those of the control animals, while the expression of *Aqp1* was 1.28-fold greater than that in the control group (Figure 5E). Surprisingly, treatment with a low dose (10^7^ CFU) of ZY-312 or with Bifico was sufficient to accelerate the recovery of *Aqp1* expression, with *Aqp1* mRNA levels in these treatment groups similar to those of the control group at day 11. In addition, the levels of *Aqp3* mRNA in the 10^9^ CFU ZY-312 treatment group showed a threefold increase compared with the control group at day 11, but were restored to control levels by day 17. Likewise, *Aqp8* mRNA levels in the 10^8^ CFU ZY-312 treatment group were increased by more than threefold on day 11, but remained high on day 17. Notably, all doses of ZY-312 increased *Aqp8* expression, while Bifico appeared to suppress the expression of both *Aqp3* and *Aqp8* compared with the control and ADD groups on day 17. In short, appropriate ZY-312 treatment could restore the expression of *Aqp1* and enhance the expression of *Aqp3* and *Aqp8*.

**Figure 6:**
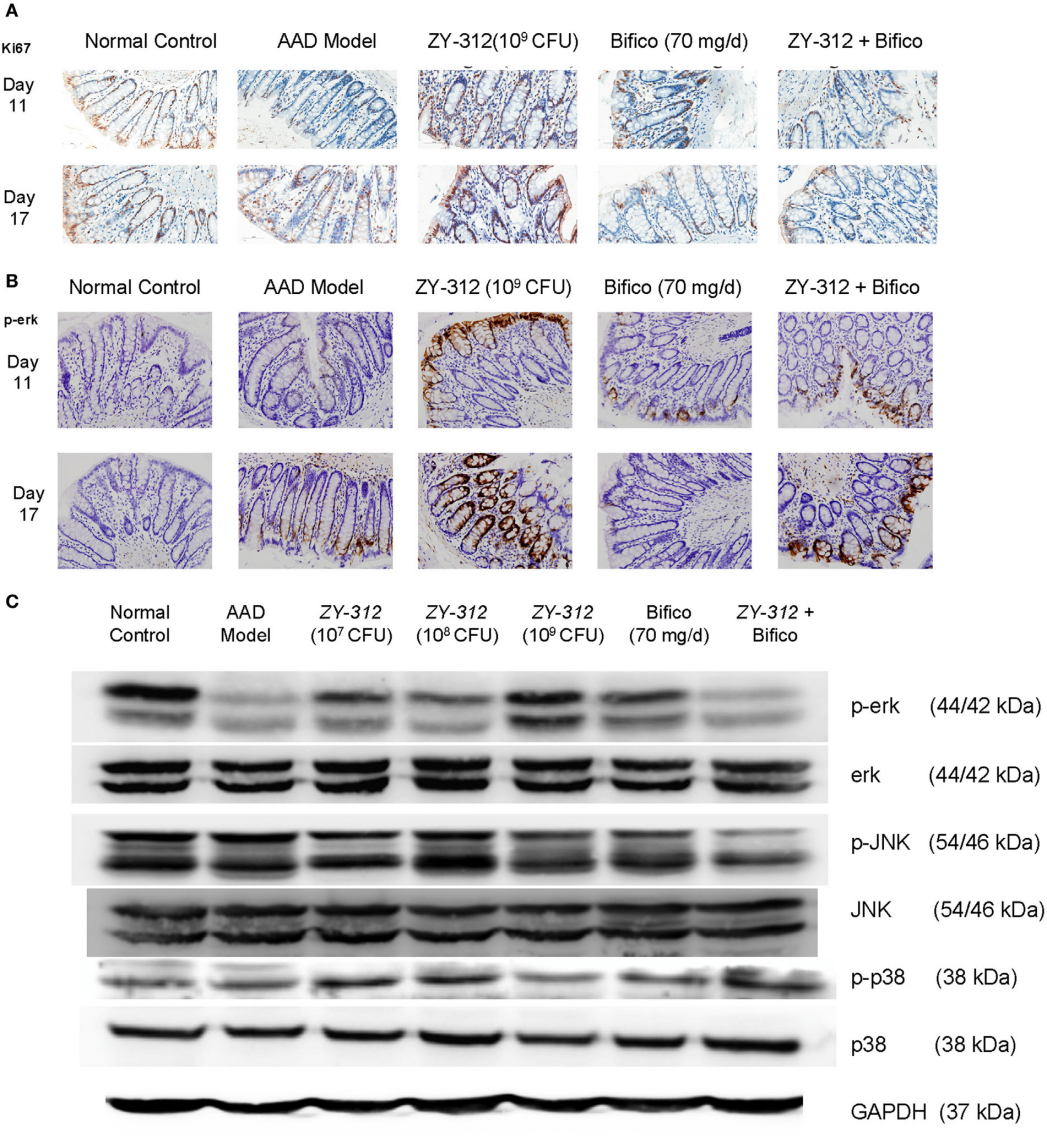
*Bacteroides fragilis* ZY-312 activates Erk signaling and promotes proliferation in antibiotic-associated diarrhea (AAD) rats. **(A,B)** Cell proliferation marker Ki67 and p-ERK1/2 were detected by immunohistochemistry in colon sections on days 11 and 17 in all groups as mentioned in Figure 5A. All images were taken at the same magnification at 40×. **(C)** Phosphorylation of extracellular signal-related kinase (ERK), c-Jun N-terminal kinase (JNK), and p38 was also detected by western blot in colon tissues on day 11 in all groups as mentioned in Figure 5A.

Finally, to further investigate whether the above changes were associated with epithelial regeneration, we stained colonic sections with Ki67, which is a cellular marker for proliferation. We found that high doses (10^9^ CFU) of ZY-312 induced a marked accumulation of Ki67-positive colonocytes along the length of the crypt and at the luminal surface (Figure 6A). Activation of mitogen-activated protein (MAP) kinase ERK is also known to stimulate proliferative gene regulatory events and initiate goblet cell differentiation (26). Our results showed ERK phosphorylation was decreased in AAD rats, while high doses of ZY-312 caused an increase in p-ERK on day 11 (Figures 6B,C). Moreover, p38 and JNK, the other two MAP kinases, which are frequently reported to trigger epithelial cellular apoptosis and inflammatory responses (27), were slightly downregulated or remained unchanged in response to high doses of ZY-312 compared with AAD group (Figure 6C).

Taken together, these results indicate that epithelial arrangement and organization are impaired in AAD rats, demonstrated by a reduction in aquaporin expression, decreased numbers of mucus-filled goblet cells, and aberrant tight junctions, all of which contribute to the impaired mucosal barrier in AAD rats. Treatment with ZY-312 plays a role in modifying the proliferation and function of the intestinal epithelium and maintaining the integrity of the intestinal barrier, thereby alleviating symptoms of AAD in rats.

## Discussion

Antibiotic-associated diarrhea is defined as unexplained diarrhea associated with antibiotic therapy (28). In the present study, we successfully established an AAD model by treating conventional rats with a mixture of antibiotics, which effectively triggered diarrhea symptoms. Previous studies suggested that the main mechanisms by which antibiotics induce AAD are the disturbance of the composition and function of the normal intestinal microbiota, and/or the allergic and toxic effects of antibiotics on the intestinal mucosa (29). We used bacterial culture methods and 16S rRNA gene sequencing to confirm that AAD rats show a significant decrease in the biodiversity of the colonic microbiota, together with overgrowth of certain genera of colitis-associated bacteria, including *Enterobacter, Klebsiella, Trabulsiella, Salmonella*, and *Escherichia*. In particular, the genera *Escherichia, Klebsiella*, and *Enterobacter* are commonly associated with nosocomial infections. A recent study has shown that multidrug-resistant strains of these three genera dominate the gut microbiota of preterm infants following specific antibiotic treatments (30). Therefore, we speculate that overgrowth of certain pathogenic bacteria may be responsible for the symptoms of AAD.

A growing body of evidence suggests that consumption of probiotics may promote gastrointestinal health, presenting a new avenue of treatment for various diseases such as inflammatory bowel syndrome, obesity, and multiple sclerosis (31–33). The commercial probiotics Bifico, which consists of *B. longum, L. acidophilus*, and *E. faecalis*, has long been considered a routine treatment for functional diarrhea in clinical practice in China (34), but its effectiveness has not been extensively evaluated. Therefore, we also administered rats with Bifico to test its efficacy in the AAD model. To our surprise, Bifico alone did not have any effect on diarrhea symptoms, nor did it worked when combined with 10^8^ CFU *B. fragilis* ZY-312, though the bacterial interaction of both remains unknown. However, an individual treatment with appropriate oral dose of *B. fragilis* ZY-312 (10^9^ CFU) is sufficient to ameliorate diarrhea symptoms in AAD rats, supported by the restoration of fecal consistency, fecal water content, and 120-min stool weight. Moreover, we found that the consumption of adequate amounts of ZY-312 could alter the composition of the intestinal microbiota in AAD rats, characterized with the inhibition of overgrown *Escherichia* and promotion of the growth of the commensal bacterium *Akkermansia muciniphila*, which has been considered as protective in type 2 diabetes (35).

With intestinal microecology being a topic of intense investigation, the modulation of *B. fragilis* in gut microbiome has become a heated issue. Generally, *B. fragilis* could be divided into NTBF and ETBF. The ETBF, which produce *B. fragilis* enterotoxin (BFT), have been found to be associated with the occurrence of diarrhea diseases including AAD in human (36). The diarrheal agent BFT could not only stimulate fluid secretion in intestinal epithelial cells *via* NF-kappaB/cyclooxygenase-2 activation but also destroy cellular interactions of intestinal epithelial cells through E-cadherin cleavage (37). What is worse, ETBF infection could also contribute to chronic colitis and promote colorectal tumorigenesis in mice (36). By contrast, NTBF act the opposite way. Recent study revealed that NTBF strain NCTC 9343 are able to colonize stably deep within crypt channels following microbiome disruption with *Citrobacter rodentium* infection or antibiotic treatment, owing to their *c*ommensal *c*olonization *f*actors (*ccf*) locus. The upregulation of *ccf* genes during *B. fragilis* strain NCTC 9343 colonization would resist colonization by the same, but not different, species, which could partially account for the rule of species-specific saturable colonization in *Bacteroides* species (38). Further, additional study demonstrated that *B. fragilis* strain NCTC 9343 can restrict enteric colonization of ETBF strain *via* a type VI secretion system (T6SS) (39). Besides, our previous study showed that *B. fragilis* strain ZY-312 can shorten the colonization time of *Vibrio parahaemolyticus* in mice, and that *B. fragilis* cell lysate enhances the ability of bone marrow-derived macrophages to phagocytize pathogenic bacteria, such as enterohemorrhagic *Escherichia coli* (40, 41). To conclude, the mocrobiota modulation of NTBF in AAD may rest in suppression of enteropathogenic bacteria together with promotion of probiotics like *A. muciniphila*.

Recent data have suggested intriguing roles for the interactions between gut microbiota and epithelial cells (42). Epithelial cells, which are sealed by tight junctions and mucus from the gastrointestinal tract, form a first line intestinal barrier between the luminal contents and the host. Therefore, these cells serve as the primary gatekeepers and regulators of bacterial interactions with the host immune system (43). Aquaporins are water-channel membrane proteins expressed in various tissues, with AQP1, 3, 4, and 8 mainly expressed in the colon (44). We observed that the AAD rats exhibited defective gastrointestinal integrity and improper epithelial organization, with decreased expression of aquaporin-encoding genes, aberrant tight junction proteins, and a decrease in the number of goblet cells compared with control animals. Although these changes have not been directly linked to pathogenic bacteria, the findings suggest that a defective gut barrier provides the opportunity for pathogens to gain access to the host, which is considered the main cause of loose stools in AAD rats. Indeed, a related study showed that *B. fragilis* strain NCTC 9343 can regulate intestinal permeability and maintain metabolic homeostasis (15). Consistent with these findings, the current study demonstrated that the administration of an adequate amount of *B. fragilis* strain ZY-312 can repair the structure of intestinal barrier by increasing the proliferation of goblet cells, which are mainly responsible for mucus production, and by enhancing the expression tight junction proteins ZO-1 and occludin, which maintain the barrier between shedding and adjacent healthy epithelial cells. Moreover, *B. fragilis* strain ZY-312 restored *Aqp1* expression in AAD rats and increased the expression levels of *Aqp3* and *Aqp8*, all of which are responsible for water transfer and absorption in the rat colon.

Various enteric microbes regulate intestinal cell tight junctions and goblet cell differentiation. Among the bacteria affected by treatment with *B. fragilis*, two have valid functions in epithelial cell organization and barrier function: *A. muciniphila* and *E. coli*. *A. muciniphila* is a mucin-degrading bacterium that improves enterocyte monolayer integrity, increases the mucus layer thickness, and corrects the gut barrier (45, 46). Moreover, *A. muciniphila* influences epithelial cell gene expression (47) and promotes mucosal wound repair *via* FPR1-dependent redox-mediated control of epithelial cell proliferation and migration (48). In addition, early-colonizing commensal *E. coli* drive the remodeling of the colonic epithelium by affecting the structure of the epithelium, mucus layer, and ion and water transport channels in rats, whereas pathogenic strains of *E. coli* bring about the opposite effects (49, 50). However, further studies are needed to elucidate whether or not these beneficial effects are exerted directly by *B. fragilis* or indirectly *via* the regulation of other bacteria.

Several pathways are associated with epithelial cell regeneration. The ERK pathway is activated by growth factors and other mitogenic stimuli, and mediates proliferative gene regulatory events and initiates goblet cell differentiation (26, 51, 52). Some commensal bacteria initiate epithelial cell growth and wound healing *via* ERK signaling (51), including *Akkermansia* species (regulated by *B. fragilis*), which stimulates ERK phosphorylation, thereby promoting epithelial wound closure and goblet cells differentiation (26, 48). These findings are in line with our present study, which indicate that a high dose of *B. fragilis* strain ZY-312, together with an increased *Akkermansia* population, induces a marked accumulation of Ki67-positive colonocytes and increased ERK phosphorylation in AAD rats. Thus, *B. fragilis* ZY-312 may play a role in modifying the proliferation and differentiation of intestinal epithelial cells *via* ERK signaling. However, further efforts are needed to investigate the specific molecular biological processes involved.

**Figure 7:**
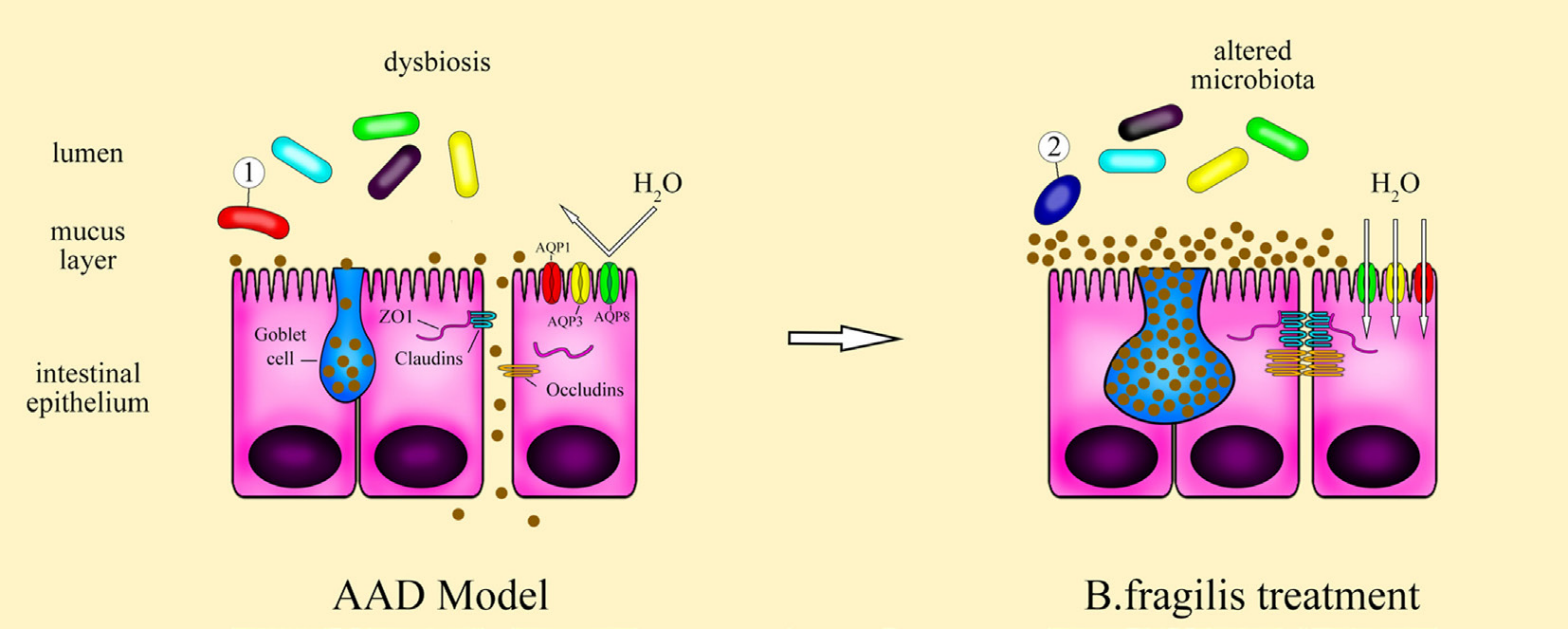
Schematic of the therapeutic role of *Bacteroides fragilis* ZY-312 in AAD symptom. Antibiotic-associated diarrhea (AAD) rats exhibit dysbiosis of the gut microbiota, with overgrowth of some pathogenic bacteria and then resulting in defective gastrointestinal integrity and improper epithelial organization with low aquaporins, aberrant tight junction proteins, and a decreased number of mucus-filled goblet cells. Treatment of AAD rats with *B. fragilis* ZY-312 alters the composition of the gut microbiota, with promotion of commensal *A. muciniphila* growth, and subsequently remodeling of the colonic epithelium by improving enterocyte monolayer integrity, increasing mucus layer thickness, and water transport in rats.

Collectively, as shown by Figure 7, these results suggest that *B. fragilis* strain ZY-312 treatment can ameliorate the gastrointestinal symptoms of AAD in rats by modulating gut microbiota, thereby restoring epithelial cell organization and barrier function, which is mediated in part through ERK signaling. Further studies are required to determine the specific roles of *B. fragilis* in the intestinal epithelium and to better understand the molecular mechanism involved. Nevertheless, this study lays a clear foundation for further investigations of microbiota-modulated epithelial function.

## Ethics Statement

All animal experiments were approved by Nanfang Hospital Animal Ethics Committee (protocol # NFYY-2014-123), in accordance with relevant ethical principles and guidelines set by the Animal Welfare Act and the NIH Guide for the Care and Use of Laboratory Animals. Experiment involving isolation of *B. fragilis* strain ZY-312 from infant fecal was approved by the Medical Ethics Committee of NanFang Hospital (NFEC-2014-040).

## Author Contributions

WZ conducted the experiments, analyzed data, and wrote the manuscript; BZ helped with performing experiments, analyzed data, and contributed to revising the manuscript; JX helped perform experiments, revised the manuscript, and contributed to data interpretation; YL and EQ analyzed data and contributed to revising the manuscript; ZhijunL did the experiments with mice; ZhengchaoL and YH analyzed data; HZ and YB directed the experiments and contributed to revise the manuscript; FZ provided overall directions and contributed to revise the manuscript.

## Conflict of Interest Statement

YL is an employee of Guangzhou ZhiYi Biotechnology Co. Ltd. The property of ZY-312 belongs to Guangzhou ZhiYi Biotechnology Co. Ltd. The use of ZY-312 in this study was supported by Guangzhou ZhiYi Biotechnology Co. Ltd. Free access to ZY-312 for research purpose would be allowed after you get the permission of Guangzhou ZhiYi Biotechnology Co. Ltd. The other authors declare no competing interest. The reviewer SS and handling Editor declared their shared affiliation.
