# Supplementary material for: Bacteroides fragilis Protects Against Antibiotic-Associated Diarrhea in Rats by Modulating Intestinal Defenses
Source: Front Immunol. 2018 May 9;9:1040. doi: 10.3389/fimmu.2018.01040 (PMC5954023; doi:10.3389/fimmu.2018.01040)
Supplement: Supplementary file 1 [file Table_1.PDF]

Table 1. Fecal consistency was assessed by a laboratory technician and classified by the following visual grading scale: (1) formed, stool maintains its shape; (2) semiformed or soft, not pour; (3) liquid, pours more easily. n=16/group before day 12, n=8/group after day 12.

Group /Day	Fecal	Day8	Day9	Day10	Day11	Day12	Day13	Day14	Day15	Day16	Day17
Normal control	1	14	16	15	15	8	8	8	8	8	8
	2	2	0	1	1	0	0	0	0	0	0
	3	0	0	0	0	0	0	0	0	0	0
AAD model	1	0	0	1	2	0	0	2	5	5	5
	2	15	15	15	12	6	6	6	3	3	3
	3	1	1	0	2	2	2	0	0	0	0
ZY-312 (10^7 CFU)	1	0	3	2	4	0	5	6	4	4	8
	2	15	12	13	12	7	2	2	4	4	0
	3	1	1	1	0	1	1	0	0	0	0
ZY-312 (10^8 CFU)	1	0	4	5	5	1	5	5	7	6	6
	2	15	9	10	9	5	2	3	1	2	2
	3	1	3	1	2	2	1	0	0	0	0
ZY-312 (10^9 CFU)	1	0	4	4	5	8	7	7	7	8	8
	2	16	11	12	11	0	1	1	1	0	0
	3	0	1	0	0	0	0	0	0	0	0
Bifico (70 mg/d)	1	0	3	3	3	4	6	4	4	5	6
	2	15	12	13	11	4	2	4	4	3	2
	3	1	1	0	2	0	0	0	0	0	0
ZY-312 + Bifico	1	0	3	7	3	5	6	5	2	4	8
	2	15	10	9	13	3	2	3	6	4	0
	3	1	3	0	0	0	0	0	0	0	0
