# Supplementary material for: Bacteroides fragilis Protects Against Antibiotic-Associated Diarrhea in Rats by Modulating Intestinal Defenses
Source: Front Immunol. 2018 May 9;9:1040. doi: 10.3389/fimmu.2018.01040 (PMC5954023; doi:10.3389/fimmu.2018.01040)
Supplement: Supplementary file 2 [file Table_2.PDF]

Table 2. Primers used for real-time PCR.

Protein	Primer sequence (forward 5′ to 3′)	Primer sequence (reverse 5′ to 3′)
AQP1	AAAGTGGCAAGGAAGGGACA	GCTGTGGATGTTGGGAAAGAG
AQP3	GAGATGCTCCACATCCGCTAC	CACACAATAAGGGCTGCTGTGC
AQP8	GGGATCTCTGGAGCCTGCATG	CTGCTGCTGTCAGAGTGGCTC
GAPDH	GCCTTCTCTTGTGACAAAGTG	TGGTGATGGGTTTCCCG
TJP1	GGAAATGTGTAAATCACCTGGAAGA	CCAAAGAACAGAAGACCACCAAC
Occludin	AATGGCATACTCCTCCAACG	AGTCATCCACGGACAAGGTC
