# Supplementary figures and images for: Bacteroides fragilis Protects Against Antibiotic-Associated Diarrhea in Rats by Modulating Intestinal Defenses
Source: Front Immunol. 2018 May 9;9:1040. doi: 10.3389/fimmu.2018.01040 (PMC5954023; doi:10.3389/fimmu.2018.01040)

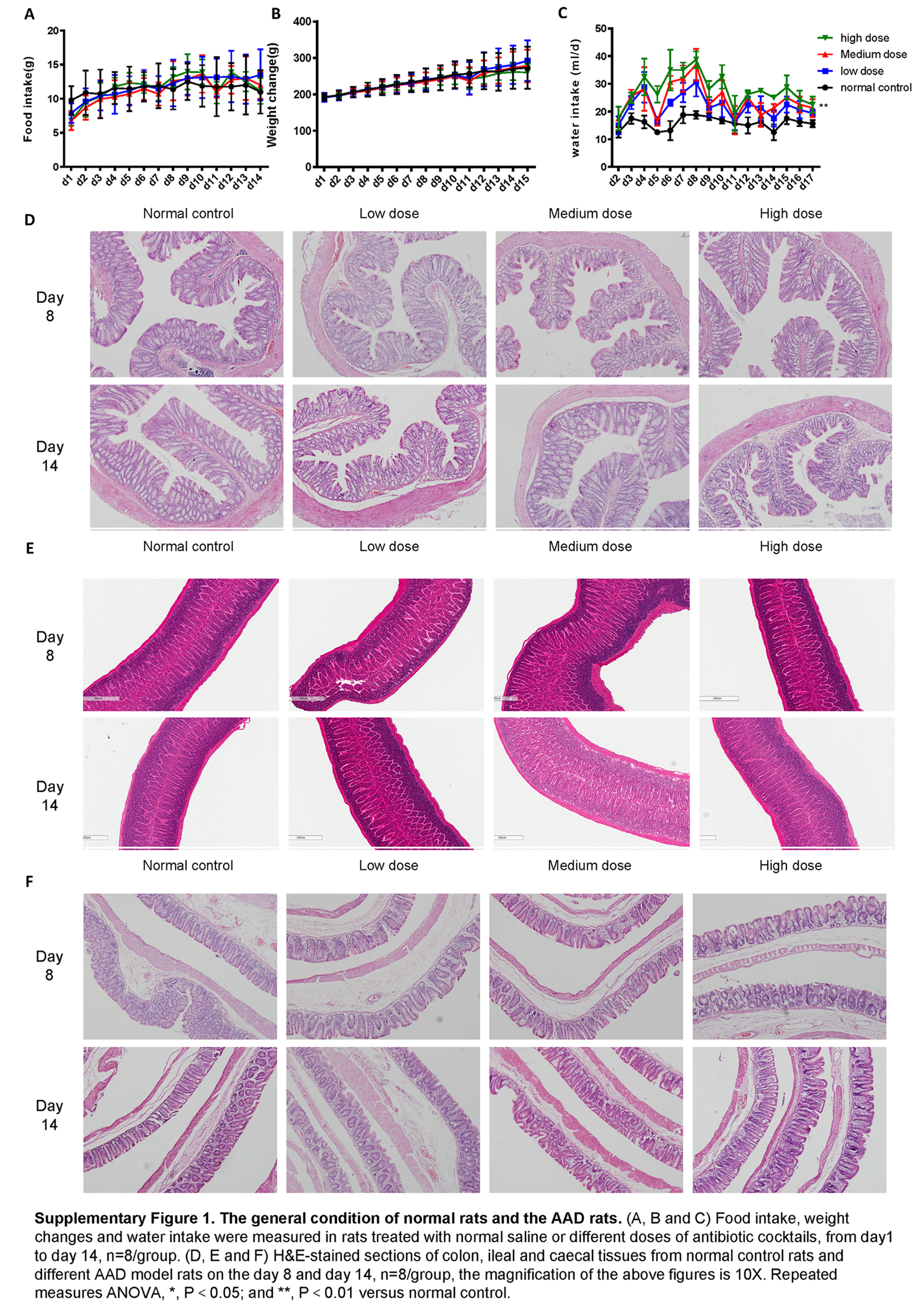

Supplement: Supplementary file 3 [file Image_1.TIFF]

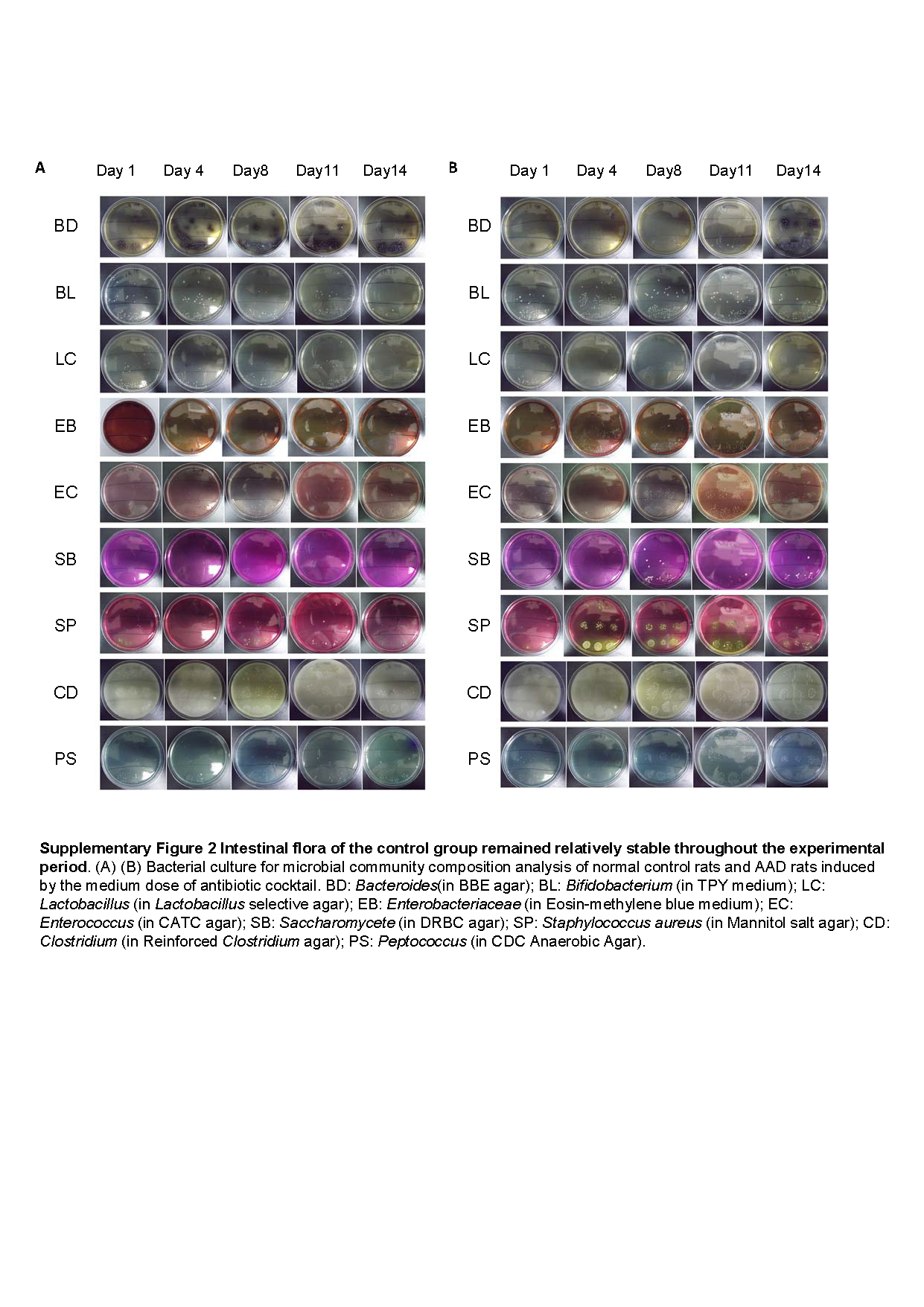

Supplement: Supplementary file 4 [file Image_2.TIFF]

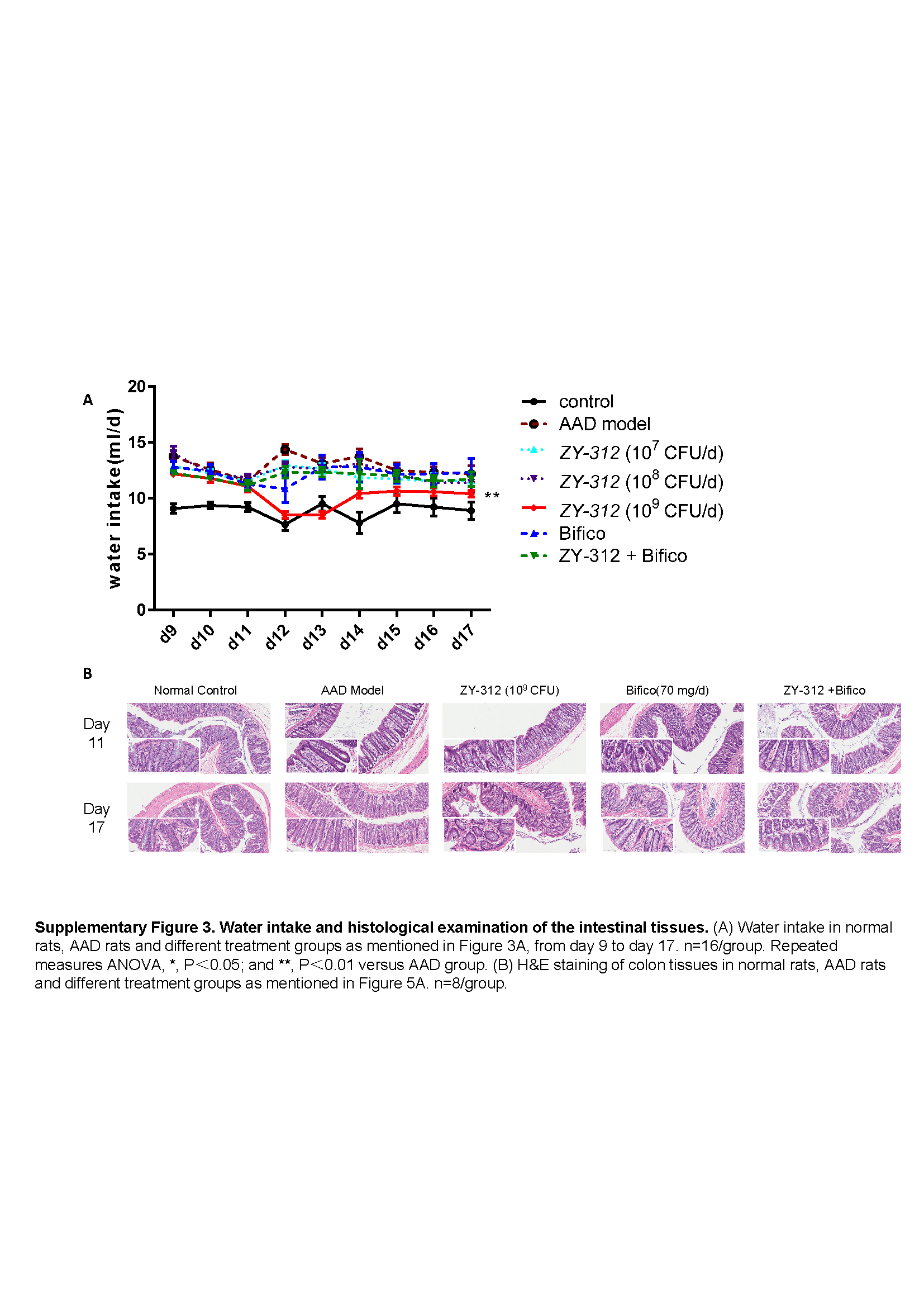

Supplement: Supplementary file 5 [file Image_3.TIFF]

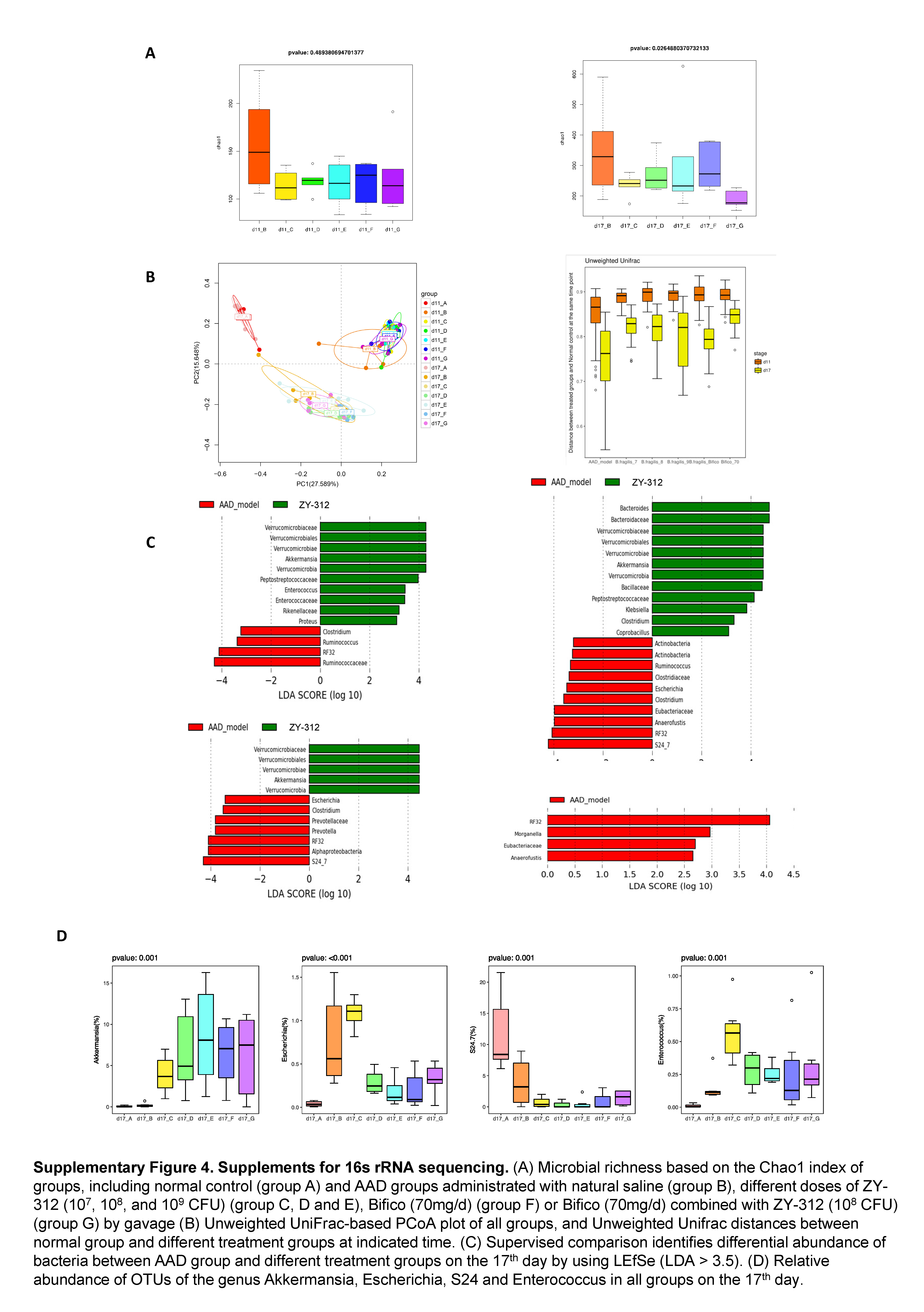

Supplement: Supplementary file 6 [file Image_4.TIFF]

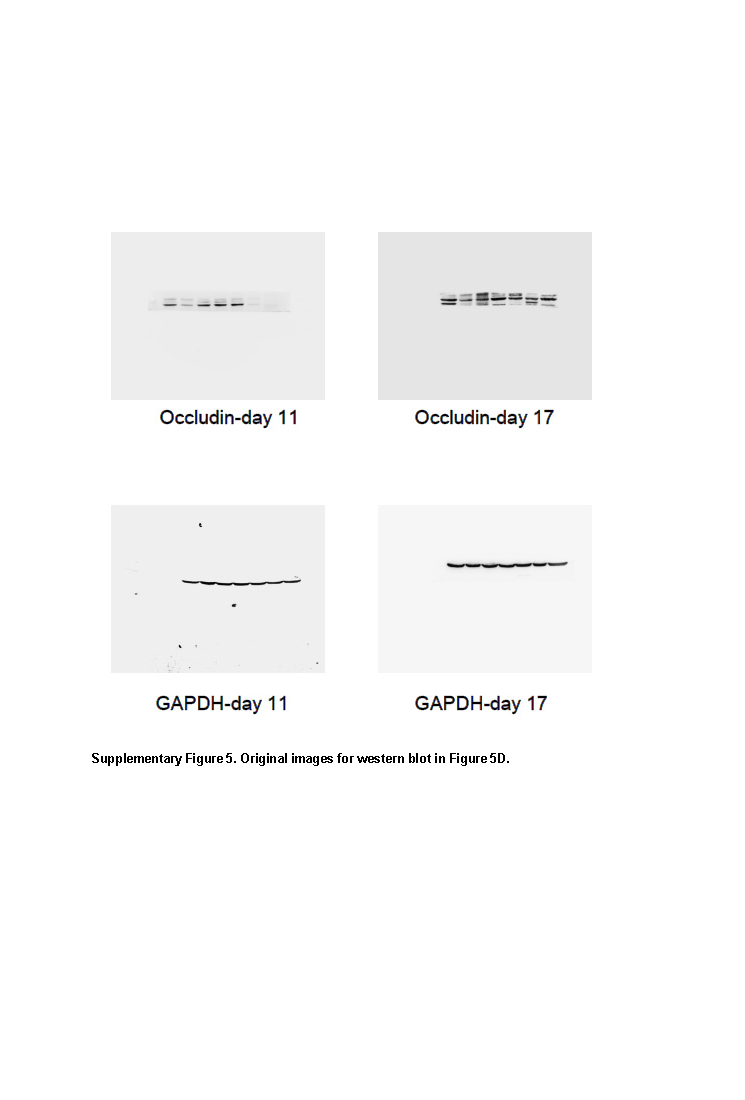

Supplement: Supplementary file 7 [file Image_5.TIFF]
